# On the Auditory-Proprioception Substitution Hypothesis: Movement Sonification in Two Deafferented Subjects Learning to Write New Characters

**DOI:** 10.3389/fnins.2017.00137

**Published:** 2017-03-23

**Authors:** Jérémy Danna, Jean-Luc Velay

**Affiliations:** Aix-Marseille Université, CNRS, Laboratoire de Neurosciences Cognitives (LNC)Marseille, France

**Keywords:** sonification, real-time auditory feedback, proprioception, compensation, motor control, handwriting

## Abstract

The aim of this study was to evaluate the compensatory effects of real-time auditory feedback on two proprioceptively deafferented subjects. The real-time auditory feedback was based on a movement sonification approach, consisting of translating some movement variables into synthetic sounds to make them audible. The two deafferented subjects and 16 age-matched control participants were asked to learn four new characters. The characters were learned under two different conditions, one without sonification and one with sonification, respecting a within-subject protocol. The results revealed that characters learned with sonification were reproduced more quickly and more fluently than characters learned without and that the effects of sonification were larger in deafferented than in control subjects. Secondly, whereas control subjects were able to learn the characters without sounds the deafferented subjects were able to learn them only when they were trained with sonification. Thirdly, although the improvement was still present in controls, the performance of deafferented subjects came back to the pre-test level 2 h after the training with sounds. Finally, the two deafferented subjects performed differently from each other, highlighting the importance of studying at least two subjects to better understand the loss of proprioception and its impact on motor control and learning. To conclude, movement sonification may compensate for a lack of proprioception, supporting the auditory-proprioception substitution hypothesis. However, sonification would act as a “sensory prosthesis” helping deafferented subjects to better feel their movements, without permanently modifying their motor performance once the prosthesis is removed. Potential clinical applications for motor rehabilitation are numerous: people with a limb prosthesis, with a stroke, or with some peripheral nerve injury may potentially be interested.

## Introduction

When someone is suffering from a loss of a given sensory modality, another preserved modality is generally used to supply equivalent sensory signals (for a review, see Bach-y-Rita and Kercel, 2003). The main trans-sensory systems were developed for blind persons, via tactile-vision substitution (e.g., Bach-y-Rita et al., 1998) or auditory-vision substitution (e.g., Renier et al., 2005). However, using the auditory modality to compensate for proprioception loss, i.e., the auditory-proprioception substitution, remains an unexplored question. To address this issue, this study proposed to assess the effects of supplementary auditory feedback in subjects having a loss of proprioception.

The proprioceptive system includes sensory signals arising from several different receptors located in different body tissues (i.e., skin, joint capsule, tendon, muscle, ligamentous, and connective tissue). The proprioceptive function is to inform about static-position and the movement of body parts. The consequences of proprioceptive loss and the question of how far vision may supplement it, has been extensively studied in sensorimotor control or adaptation in deafferented subjects (Lajoie et al., 1992; Ghez et al., 1995; Sainburg et al., 1995; Nougier et al., 1996; Krakauer et al., 1999; Scheidt et al., 2005; Pipereit et al., 2006; Sarlegna et al., 2006, 2010). Without proprioception, subjects are very reliant on vision, and can show near normal performance in visual tasks such as visual reaching. In a mirror-drawing task, whereas healthy participants pay attention to controlling the incongruent information provided by visual and proprioceptive feedback, deafferented subjects have less difficulty adapting their movement because the sensory conflict does not exist for them (Lajoie et al., 1992). However, their performance is very much affected in tasks performed without continuous visual information (Fourneret et al., 2002) or in tasks requiring adaptation in musculoskeletal dynamics for which proprioceptive feedback is critical (Sainburg et al., 1995; Krakauer et al., 1999).

Interestingly, the impact of proprioceptive loss on motor learning has mainly been studied in adaptation tasks but, to the best of our knowledge, never during the motor learning of a new pattern under normal, non-biased, perceptual conditions. When a new pattern is learned, the task-relevant sensory information, provided from both the environment and the body, are integrated to allow the fluent execution of the pattern and its memorization as an internal model (for a review, Wolpert et al., 2011). At the beginning, the learners do not have a kinesthetic reference for the movement and hence they should control it more visually. In arm control for example, hand trajectories would initially be planned in spatial coordinates without taking account of the joint motions (Morasso, 1981).

Until now, theories on motor control and learning have mainly focused on the role of vision and proprioception, considering the movements as silent. Yet, human actions often generate sounds whose variations directly inform about the movements made. When these sounds are systematically present during motor learning, strong audio-motor associations are created in such a way that, after learning, the sounds alone will evoke the movement, and reciprocally the silent movement will recall its associated sound (Kohler et al., 2002; Zatorre et al., 2007). Consequently, when movements are naturally silent, adding auditory information during motor execution may improve their control and thus facilitate memorization (e.g., Effenberg et al., 2016). The method, so-called movement sonification, consists of translating, in real-time, some movement parameters into synthetic sounds. The multimodal (visual, auditory, and proprioceptive) integration of sonified movements has been shown to be effective in motor control and learning (see Sigrist et al., 2013 for a review). Applications are various, from sports practice (e.g., Effenberg, 2005), clinical rehabilitation (e.g., Scholz et al., 2014), to school education (e.g., Danna et al., 2014).

Handwriting is particularly relevant for evaluating auditory-proprioception substitution. Despite the faint scratching of the pen, handwriting is considered as a silent activity, mainly controlled by vision and proprioception (for a review, see Danna and Velay, 2015). Handwriting is possible without proprioceptive feedback. Provided vision was available, the quality of the written trace of a deafferented subject was comparable to that of control subjects (Teasdale et al., 1993). Nevertheless, when comparing a deafferented subject (one of the two subjects) and control subjects, though the words written by the former remained legible, the kinematics of her handwriting movement were deeply affected (Hepp-Reymond et al., 2009). To conclude, vision and proprioception are complementary in handwriting control: spatial information about the static trace is mainly provided by vision and movement information is mainly provided by proprioception (Danna and Velay, 2015).

For these reasons, we decided to sonify handwriting for auditory-proprioception substitution. Providing spatial information by the means of sounds is not a relevant strategy, because spatial information is already well supplied by vision. The purpose was rather to translate information about the movement, usually provided by proprioception, into auditory information. In particular, velocity signals, mainly provided by muscle spindles (Cordo et al., 2011), play a crucial role in movement perception and control when precise and fluid movement is required. So the question was, how to sonify handwriting velocity? Actually, when listening carefully to the sound produced during handwriting, a friction sound generated by the pen-paper interaction can be heard (Thoret et al., 2014) compared this real friction sound to a synthetic friction sound whose timbre was related to the pen velocity and they observed that this velocity sonification adequately informed about the pen displacement. They concluded that velocity sonification enters into a natural mapping between the sound and the action in order to contribute to the building of a multimodal sensorimotor representation of handwriting (Thoret et al., 2014). Based on this assumption, a similar sonification strategy was tested and validated for handwriting assessment (Danna et al., 2015a), handwriting learning (Danna et al., 2015b), and the rehabilitation of dysgraphia (Danna et al., 2014).

The auditory-proprioception substitution hypothesis was proposed as a strategy for stroke rehabilitation (Scholz et al., 2014) but we have yet to see the experimental validation of this hypothesis (Ghez et al., 2000) made an encouraging pilot study but without actual control experiments. The purpose of the present experiment was thus to assess the auditory-proprioception substitution in both two deafferented subjects and 16 control participants who all had to learn new characters with and without associated sonification.

Four predictions could be made before the experiment:

- Because the present strategy consists in translating velocity information into sounds, we predict that training with sonification will improve handwriting kinematics and not spatial accuracy of the written trace (as already observed by Danna et al., 2015b).
- The loss of proprioception is known to affect handwriting kinematics (Hepp-Reymond et al., 2009), therefore training with sonification will have a larger effect in deafferented than in control participants.
- If the supplementary auditory signals only help to control the movement during training, but are not integrated within an internal multisensory model of the character, then applying sonification during training will only have a short-term effect.
- Conversely, if the auditory signals are actually incorporated into the internal model of the character, then they will improve the kinematics of character production at longer term.

## Methods

### Participants

Two deafferented subjects, GL (right-handed female, 65 years) and IW (left-handed male, 61 years), participated in the experiment. The Edinburgh Inventory (10-item version, Oldfield, 1971) conducted by Lefumat et al. (2016) revealed a Laterality Quotient of +77% for GL and −100% for IW. Both suffer from a complete loss of touch, vibration, pressure and kinesthetic senses below the neck in IW and below the nose in GL (Cooke et al., 1985; Cole and Sedgwick, 1992). The sural nerve biopsy conducted by Cooke et al. (1985) on GL revealed that fibers larger than 6.5 μm represented only 1.6% of the total number of fibers. However, both subjects have perceptions of pain and temperature, indicating a selective impairment of the large diameter peripheral sensory myelinated fibers. Motor fibers are not affected as shown by motor nerve conduction velocities and needle electromyography investigation of the arm muscles. H-reflexes are absent, no sensory nerve action potentials can be registered in the arms, and no cortical response can be evoked by electrical stimulation of the peripheral nerves of either arm. GL has been suffering from a permanent and specific loss of the large peripheral myelinated sensory fibers since she was 31 (for more details about her history and disease characteristics, see Cole and Paillard, 1995). IW experienced a permanent and specific loss of the large peripheral myelinated sensory fibers when he was 19 (for more details about his history and disease characteristics, Cole and Sedgwick, 1992; Cole and Paillard, 1995).

Sixteen healthy, age-matched control subjects (8 right-handed women and 8 men, between 58 and 68 years) volunteered for the experiment. Two of the controls were left-handed. None of the controls reported any relevant medical history. This study received a prior approval from the Ethics Committee of Aix-Marseille University and the CNRS (N° RCB 2010-A00155-34). All participants signed a written informed consent before starting the experiment, in accordance with the ethical standards set out in the Declaration of Helsinki.

### Task

**Figure 1:**
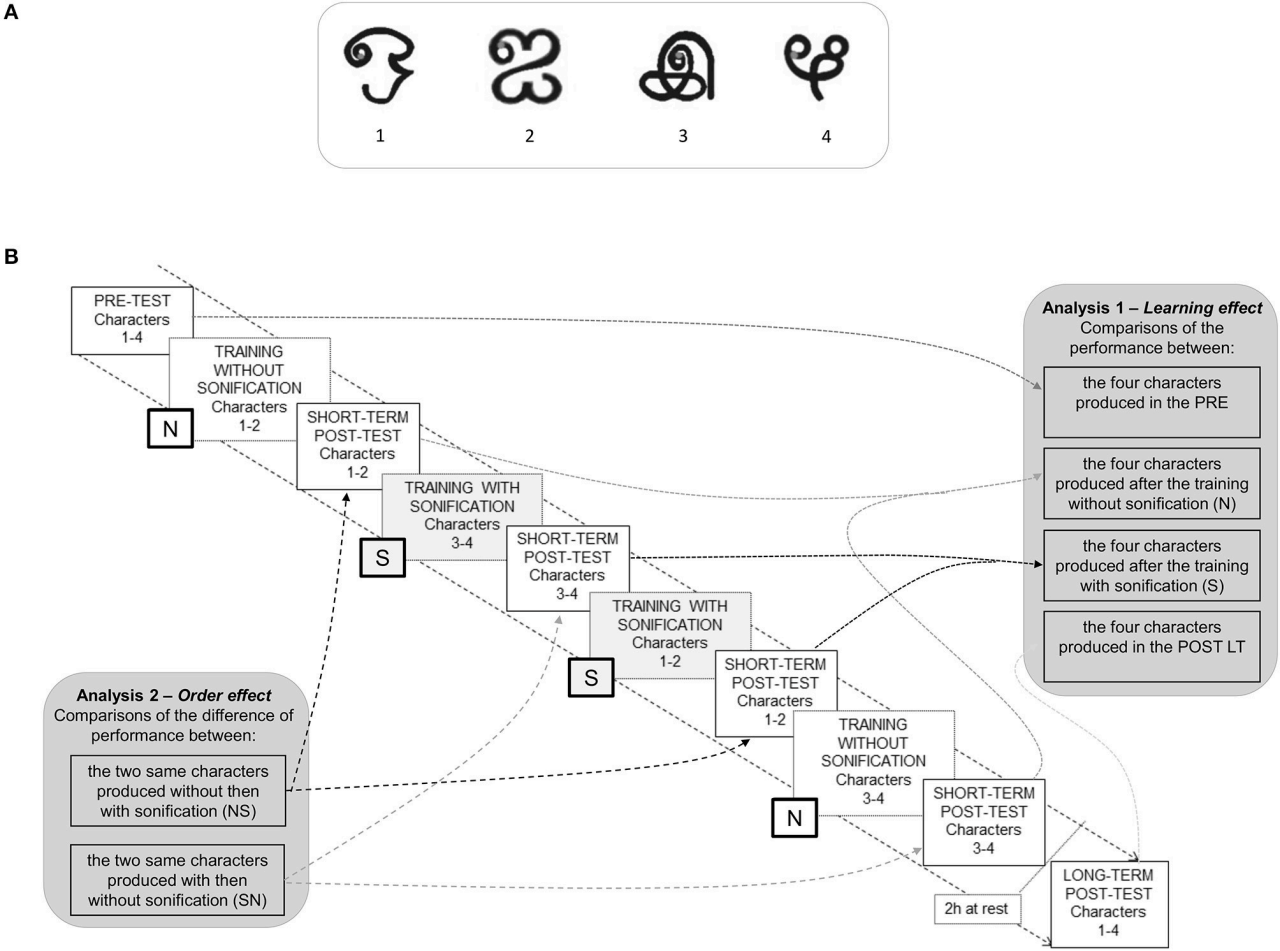
**(A)** Illustration of the four learned characters. **(B)** Description of the experimental design and analyses. Each character was trained without sonification (S) and with sonification (N). The presentation order of characters was counterbalanced between participants (see text).

The task consisted of learning to write four new characters (Figure 1A) on a sheet of paper (A4 format: 21.0 × 29.7 cm) affixed to a graphic tablet (Wacom, Intuos3 A4, sampling frequency 200 Hz) using an ink pen. The characters were extracted from the Tamil script. Character 4 was slightly modified in order to be drawn without lifting the pen. Each character was presented at the top of the sheet. A gray point has been added to indicate the starting point on the characters. A square (4.0 × 4.0 cm) was drawn for each repetition in order to produce a character of comparable size.

### Procedure

The experiment began with a short familiarization during which participants were asked to draw some simple geometric shapes with the auditory feedback in order to become informed about the meanings of the sonification. For the sake of clarity, the term “sonification” will be used rather than auditory feedback. The actual experimental design included a pre-test, a training session, and two post-tests. The first post-test (POST ST) was performed just after the training session of each character, and the second about 2 h later (POST LT). The pre-test and the two post-tests were exactly the same: The participants wrote each of the four characters once without sonification.

We used a classical within-subject ABBA protocol consisting of two different sessions (A and B) repeated in a different order. More precisely, the four characters were learned by pairs in two modes of training, one without sonification (session N) and one with sonification (session S), respecting the NSSN protocol. During the training sessions, the participants wrote each of the two characters 16 times. Two characters (characters 1 & 2) were learned first without, then with, sonification (order NS) and the other two (characters 3 & 4) were learned first with, then without, sonification (order SN; see Figure 1B). The order of characters written with sonification was counterbalanced between the two deafferented subjects and between controls in such a way that half of participants began the training sessions with characters 1–2 and the other half began with characters 3–4. Participants were asked to draw the characters in a single movement, without lifting the pen from the gray starting point to the end of the character.

### Sonification strategy

We applied the same sonification strategy already used in a previous study (Danna et al., 2015b), with the exception of impact sounds which were not present here. Sonification was generated in real time with Max software (http://cycling74.com). An example of sonified handwriting is available online in the Supplementary Material (Supplementary Video File 1).

A rubbing sound was associated to a correct handwriting velocity. This synthetic sound was close to the sound generated by writing with chalk on a blackboard. Technically, the synthesis was based on a source-resonator model which simulates the physical sound source as the result of successive impacts of a pencil on the asperities of a given surface. The surface roughness is modeled by a noise reflecting the height of the surface asperities while the velocity profile of the pencil is modeled by low-pass filtering the noise with a time varying cutoff frequency that creates timbre variations according to the velocity profile (for more details, Conan et al., 2014).

When handwriting was too slow, the *rubbing* sound changed into *squeaking* sound. These squeaking sounds were based on non-linear (stick–slip) friction behavior (for more details, Thoret et al., 2013). This strategy is drawn from the metaphor of the squeaking of a door which naturally leads the writers to increase their movement speed in order to avoid this unpleasant noise. The synthesis model enabled sudden transitions between squeaking sounds and rubbing sounds. Transitions from the friction sound to the squeaking sound occurred when the instantaneous tangential velocity dropped below 1.5 cm s^−1^ (Danna et al., 2015b).

Finally, the pen pressure on the paper sheet, a measure directly provided by the tablet, was linearly associated to the sound volume, so that the greater the pen pressure the higher the volume.

### Data analysis

**Figure 2:**
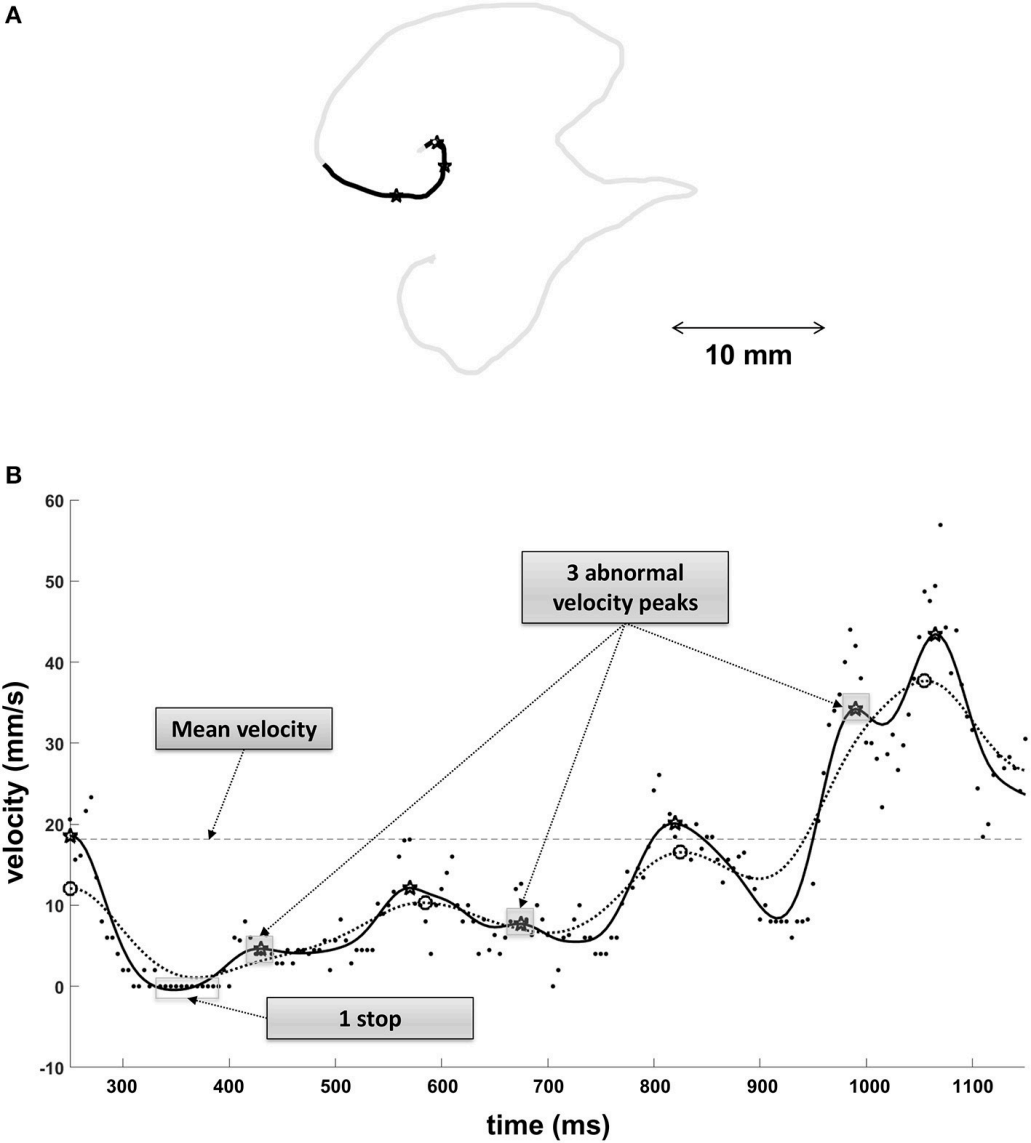
**(A)** Illustration of a character written by GL. **(B)** Velocity profile from a portion of the written character (in black). The three kinematic variables are illustrated: The dotted line corresponds to the mean velocity. The three black stars correspond to the abnormal velocity peaks which are present only when filtering the velocity with a cutoff frequency at 10 Hz (black line) but not when filtering the velocity with a cutoff frequency at 5 Hz (dotted curve). Finally, the white rectangle corresponds to a stop (white dot in **A**) determined by a null (raw) velocity longer than 35 ms.

Four variables, three kinematic and one spatial, were computed from the (x,y) position of the pen on the tablet. The kinematic analyses were illustrated in Figure 2.

The movement velocity was the mean translational velocity from the starting point until the final lift, when the character was completed.

The number of abnormal velocity peaks was determined by the Signal-to-Noise velocity peaks difference (SNvpd). SNvpd is the difference between the number of velocity peaks after filtering the tangential velocity with a frequency cutoff (fc) of 10 Hz and the number of velocity peaks after filtering the tangential velocity with an fc of 5 Hz (Danna et al., 2013). Accordingly, the number of abnormal velocity peaks is an index of movement fluency: the less fluid the movement, the greater the number of abnormal velocity peaks and *vice versa*.

The number of stops was determined by counting the moments when the pen stopped during the drawing of the character. Note that stops are distinct from lifts of the pen: the former occurred even though the pen was still in contact with the paper. Stops shorter than 35 ms were considered as normal stops (Paz-Villagrán et al., 2014). Therefore, here we only took into account those longer than 35 ms. Because the task consisted of writing the characters without lifting the pen, we assume that more stops were produced at the beginning, when participants had not yet memorized the characters and had to look at the model.

The spatial accuracy was determined by the Dynamic Time Warping (DTW) distance. DTW distance is the measurement of the spatial error between the character written by participants and a character prototype considered as a reference. More precisely, it corresponds to a point-to-point comparison between the two characters for which both spatial and temporal information is available. The DTW distance is computed as the average Euclidean distance between all pairs of matching points (for more details about criteria used for matching, see Niels et al., 2007). The character prototypes were realized by a proficient adult who practiced writing each character with the aid of a model until the perfect shape was achieved. The series of (x,y) coordinates corresponding to the shape of each character were then filtered with a 4th order low-pass Butterworth filter with a fc of 5 Hz. These four characters were considered as “ideal” characters and the greater the disparity between them and the character drawn by a subject, the greater the DTW distance. For the sake of clarity, we took the inverse of DTW distance as an index of spatial accuracy: The better the character matched with the reference, the higher the score.

### Statistics

Statistical analyses were conducted in two steps.

(1) *Learning effect*. As can be seen in Figure 1B (right), the effect of practice was assessed for the control group by computing the mean performance for the four characters written in the pre-test (*PRE*), those written just after the training session with sonification (*POST ST—after S*), those written just after the training without sonification (*POST ST—after N*), and those written about 2 h afterwards (*POST LT*). These data were submitted to an analysis of variance (ANOVA) with the four Learning conditions as repeated measures and Bonferroni's post hoc tests when necessary. To compare GL's & IW's data to those of controls, we used *t*-test comparisons of a single value to a population sample (Nougier et al., 1996; Sarlegna et al., 2010) for the four learning conditions. The significance threshold was corrected to 0.0125 for the four *t*-test comparisons (Bonferroni's correction).

(2) *Order effect*. Within-subject ABBA protocol induces an order effect because some characters learned without sonification were learned after some characters learned with sonification. To evaluate the order effect, we averaged the performance in the same pairs of characters under the four conditions N, S, S, and N in the short term post-tests (Figure 1B—left). Then, we computed the difference of performance between the post-test of characters learned first with then without sonification, taking into account the presentation order, namely SN (without then with sonification) versus NS (with then without sonification). A difference significantly above or below zero revealed an effect of sonification and the order effect appeared if the difference in performance was observed in the NS order only. For that, we used *t*-test comparisons of a single value (0) to the controls' performance with Bonferroni's correction for the two presentation orders (significant threshold at 0.025). In order to assess whether sonification had a greater effect in deafferented subjects than in the controls, we also used *t*-tests comparisons of a single value to a population sample (with Bonferroni's correction) to compare the differences in performance of the controls to those of the deafferented subjects.

## Results

The effects of learning and sonification are presented in turn on each of the four variables analyzed.

### Learning effect

**Figure 3:**
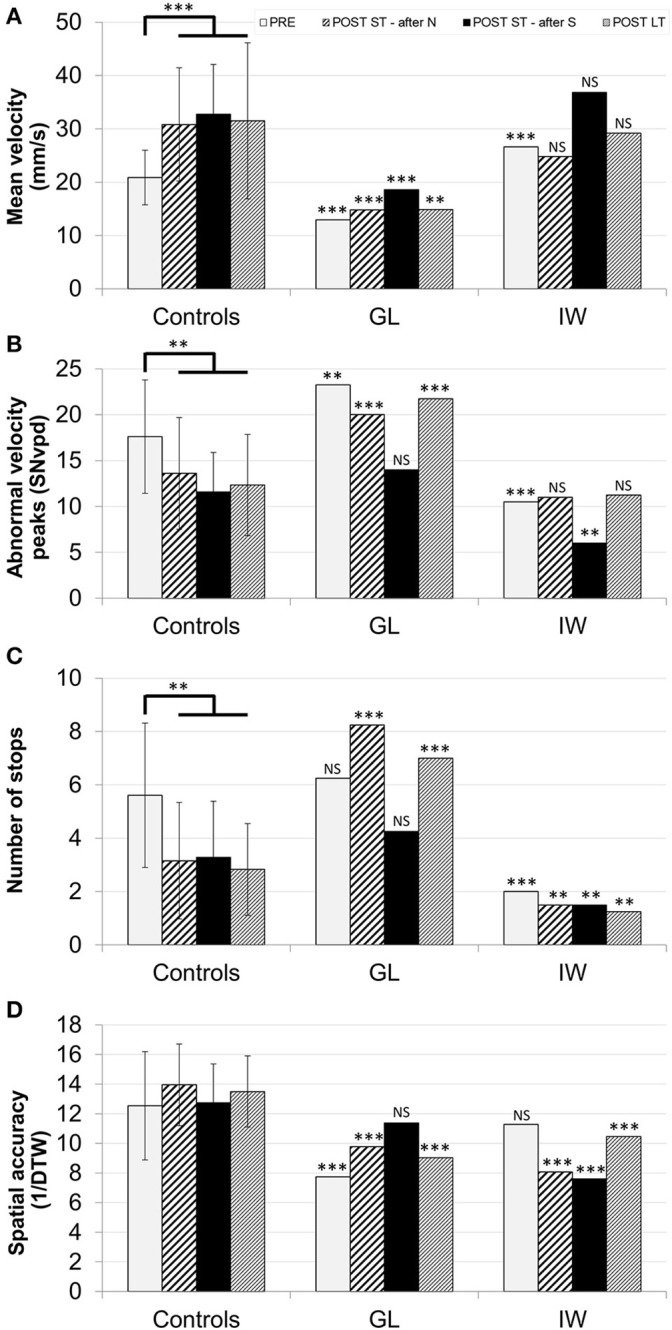
**Mean performance of the control group, GL and IW in the pre-test (*PRE*), the post-test following the learning sessions without sonification (*POST ST—after N*), the post-test following the learning sessions with sonification (*POST ST—after S*), and the post-test at T0 + 2 h (*POST LT*)**. Error bars correspond to between-participants SD of the control group. ^**^p < 0.01; ^***^p < 0.001. The performance was assessed with the mean velocity **(A)**, the abnormal velocity peaks **(B)**, the number of stops **(C)**, and the spatial accuracy **(D)**.

**Figure 4:**
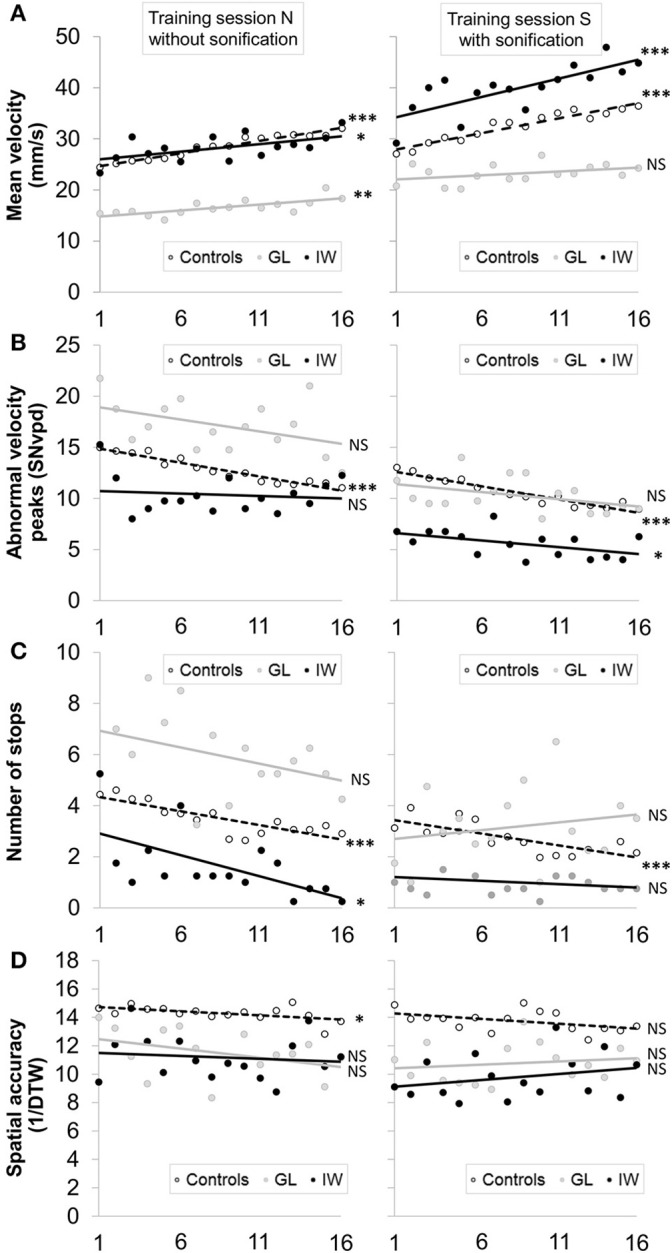
**Evolution of performance of the control group, GL and IW throughout the 16 trials during the training sessions N without sonification (left) and the training sessions S with sonification (right)**. Trend lines correspond to simple linear regressions. The performance was assessed with the mean velocity **(A)**, the abnormal velocity peaks **(B)**, the number of stops **(C)**, and the spatial accuracy **(D)**.

The performance of control and deafferented participants in the four learning tests without sonification are presented in Figure 3. Illustrations of the characters produced by GL and IW are supplied in the Supplementary Material (Supplementary Figure 1). Finally, the performance of control and deafferented subjects during the training phases with and without sonification are presented in Figure 4.

#### Movement velocity

The control group exhibited a main effect of learning, *F*_(3, 45)_ = 15.24, *p* < 0.001, $\eta_{p}^{2}$ = 0.50 (see Figure 3A). Bonferroni's *post-hoc* tests confirmed that the mean velocity in the three post-tests was higher than in the pre-test (*p* < 0.001). The comparison between the three post-tests was not significant.

The comparison between GL, IW, and controls revealed that GL was always slower than the controls (*p* < 0.001 for the four comparisons) whereas IW's velocity was comparable to that of the controls except in the pre-test where it was even higher (*p* < 0.001). Moreover, contrary to the controls, both GL's and IW's velocities in the *POST ST—after N* and in the *POST LT* were not different from their initial velocity in the *PRE* (see Figure 3A).

Figure 4A shows the evolution of velocity across the 16 repetitions within the training sessions (without and with sonification). When comparing the evolution of the velocity (trend lines), two observations can be made: (1) comparing the Y-intercept between sessions N and S gives an idea of the initial effect of sonification at the first trial, before learning. (2) comparing the slopes between sessions N and S informs about the sonification effect on learning progress over the 16 repetitions. In control participants, adding sounds during training (session S) gave rise to a slight increase in writing speed (Y-intercept) but did not change the learning progression (identical slopes). GL was globally slower than the controls, but she benefited more than them from the presence of sonification at the first trial (Y-intercept). However, her learning slope was not modified by the sonification (null slope/non-significant regression in the session S). IW was quite similar to the controls without sonification (session N), however with the sonification (session S) both his initial speed and learning progression were greater than in control condition.

#### Number of abnormal velocity peaks

The control group exhibited a main effect of learning, *F*_(3, 45)_ = 11.35, *p* < 0.001, $\eta_{p}^{2}$ = 0.43. Bonferroni's *post-hoc* tests confirmed that the number of abnormal velocity peaks in the three post-tests was lower than in the pre-test (*p* < 0.01, see Figure 3B). The two short-term post-tests were not different in the control group whatever the order of the training sessions they had followed.

As can be seen in Figure 3B, GL produced more abnormal velocity peaks than the control group in the *PRE*, the *POST ST—after N*, and the *POST LT* (*p* < 0.01 for the three comparisons), but not for the *POST ST—after S*. In other words, GL generally wrote the characters less fluently than the control participants, except when she had just learned the characters with sonification. The sonification effect on GL's movement fluency was larger than that on control participants.

IW wrote the characters with less abnormal velocity peaks than the control participants in the *PRE* and when he learned to write the characters with sonification (*p* < 0.01 for the two comparisons) but neither in the *POST ST—after N* nor in the *POST LT*.

Contrary to the control group, the movement fluency of GL and IW in the post-test of characters learned without sonification and in the post-test at T0 + 2 h were almost identical to their initial performance in the pre-test.

When comparing the evolution of the abnormal velocity peaks during the two modes of training in the control group (Figure 4B), a slight initial effect of sonification at the first trial (Y-intercept) was noted but no impact on learning progression (identical slopes) can be observed. Regarding GL, whereas she wrote the characters less fluently than the controls without sonification (with a great variability across repetitions), she performed close to the controls with sonification. However, due to her variable performance, the regression analysis did not reveal a significant evolution. Finally, IW was more fluent from the very beginning than the controls (lower Y-intercept) but he experienced no improvement across the 16 trials. With sonification, a positive effect at the first trial (Y-intercept) was noted, as well on his learning progression.

#### Number of stops

The control group exhibited a main effect of learning, *F*_(3, 45)_ = 8.51, *p* < 0.001, $\eta_{p}^{2}$ = 0.36. The Bonferroni's *post-hoc* tests confirmed that the number of stops in the three post-tests was lower than in the pre-test (*p* < 0.01, see Figure 3C). The results of the comparison between the two short-term post-tests were not significantly different.

The deafferented subjects and the control groups all produced different results. GL had a significantly greater number of stops than the controls for the *POST ST—after N* and for the *POST LT* only (*p* < 0.001 for both comparisons, Figure 3C). During the *PRE* and the *POST ST—after S*, her stops number was comparable to controls. In other words, as for movement velocity and fluency, her stops numbers in the post-test of characters learned without sonification and in the *POST LT* were almost identical to her initial performance. This was not the case for the control group. Concerning the comparison of stops number between IW and the control group, the difference was significant for all tests. Even before learning, IW had seldom stopped during his movements, likely because he was using a different control strategy.

Regarding the evolution of the number of stops across the repetitions within the two modes of training (Figure 4C), the same observations as for abnormal velocity peaks can be made in the controls. Concerning GL, sonification allowed her to perform the task with a mean number of stops comparable to that of the controls but no learning progression was observed, whatever the training mode (N or S). IW produced very few stops (between 0 and 2, except at the first trial in the training session N), suggesting a feedforward control strategy.

#### Spatial accuracy

In the control group, the spatial accuracy did not evolve from the pre-test to the long-term post-test, *F*_(3, 45)_ = 1.07, NS (see Figure 3D). GL drew the characters with a lower accuracy, except the characters which had been learned with sonification. Contrary to GL, IW displayed a spatial accuracy close to that of the control participants in the pre-test only. In all the post-tests, whatever the training mode, he showed a lower spatial accuracy than the control group, irrespective of the presence of sonification (see Figure 3D).

Comparing the evolution of spatial accuracy between the two training sessions indicates a slight effect of sonification on the performance variability in the control group (Figure 4D). In both GL and IW, 1- their spatial accuracy was lower than that of the controls, and 2- sonification had a slight negative effect on spatial accuracy (observed by a low Y-intercept in sessions S) but no effect on learning (null slope/non-significant regression in the sessions N and S).

### Order effect

As explained, our within-subject NSSN protocol induces an order effect. Differences of performance between the two POST ST (after S and after N) were thus computed in each order (NS vs. SN) and reported in Table 1.

**Table 1:** **Mean difference of performance (between-participants SD) between the post-test when characters were learned with sonification and the post-test when characters were learned without sonification (S-N) according to the order of presentation (NS: first without then with sonification vs. SN: first with then without sonification) for the control group, GL and IW**.

		**NS order: first without then with sonification**	**SN order: first with then without sonification**
Difference in velocity	GL	8	−0.4
	IW	***19.3***	***4.7***
	controls	**6.7 (±6.2)**	−2.9 (±7.2)
Difference in velocity peaks	GL	**−*8.5***	−3.5
	IW	**−*11***	1
	controls	**−3.2 (±3.5)**	−0.8 (±6.6)
Difference in stops number	GL	**−*2.5***	**−*5.5***
	IW	−1.5	1.5
	controls	−0.4 (±2.5)	0.7 (±3.75)
Difference in spatial accuracy	GL	***6.9***	−3.7
	IW	−2.5	***1.6***
	controls	−1.3 (±3.3)	−1.1 (±3.7)

*In bold: significant difference revealed by comparing the control group to zero (analysis 3). In bold and italic: significant difference revealed by comparing the control group to GL and to IW (analysis 4)*.

#### Movement velocity

In the control group, comparing the characters learned with versus without sonification (S-N) revealed that the velocity difference was significant in NS order (*p* < 0.001) but not in SN order (*p* = 0.13, see Table 1). As expected, this marked difference between the training orders suggests that when two characters were first learned with sonification, the gain in velocity was maintained afterwards when two new characters were trained without sonification.

Does sonification have a greater effect on deafferented subjects than on the control group? Results revealed that, irrespective of the order (NS vs. SN), the difference of velocity between the post-test of characters learned with versus without sonification (S-N) was greater for IW than for the controls (see Table 1). Note that this was not the case for GL whose spontaneous velocity was much lower than that of IW and the control group.

#### Number of abnormal velocity peaks

In the control group, comparing the fluency difference when characters were learned with vs. without sonification (S-N) revealed that the difference was significant in NS order (*p* < 0.01) but not in SN order (*p* = 0.63, see Table 1).

Does sonification have a greater effect on deafferented subjects than on the control group? Results showed that in NS order, i.e., when the characters were learned first without and then with sonification, the difference in abnormal velocity peaks between the two training sessions was larger in the deafferented subjects than in the control participants (see Table 1). In the reverse SN order, the difference of fluency was not significantly greater than in the control participants. This marked difference between the two orders of training suggests that, both in control and deafferented participants, the fluency increased following the training with sonification and stayed high, even though the subsequent characters were trained without sonification.

#### Number of stops

In the control group, comparing the fluency of the characters learned with versus without sonification (S-N) revealed that, irrespective of the order, the difference was not significant (*p* = 0.49 for NS order and *p* = 0.47 for SN order, see Table 1). Therefore, the number of stops was not influenced by sonification in the control group.

Whatever the order (NS vs. SN), the difference in the number of stops was greater for GL than for the control group. This was not true for IW who made few stops, whatever the learning task or the sonification condition.

#### Spatial accuracy

In the control group, comparing the characters learned with versus without sonification (S-N) revealed that, irrespective of the order, the difference in spatial accuracy was not significant (*p* = 0.13 for NS order and *p* = 0.24 for SN order, see Table 1). These results confirmed that spatial accuracy was not influenced by sonification in the control group.

The increase in spatial accuracy was significantly greater in GL than in the control participants in the NS order only. IW's spatial accuracy was slightly greater than in the control participants in the reverse SN order (see Table 1).

## Discussion

The goal of this study was to evaluate auditory-proprioception substitution in two persons lacking proprioception. The effects of real-time auditory feedback were assessed during the motor learning of new graphic patterns. The results of this experiment can be summarized as follows:

### In control participants

Overall, control participants were able to learn the new characters without sounds, but the sonification improved their learning: characters learned with sonification were reproduced more quickly and more fluently than those learned without. In other words, adding auditory kinematic signals during training lead to an improvement of kinematic variables when the characters were subsequently drawn without the sounds. These results are in agreement with those of a previous study where participants had to learn new characters with their non-dominant hand (Danna et al., 2015b). The improvement was present in the short term, but it was also observed in the longer term, 2 h after the end of the training sessions. However, this motor improvement was not accompanied by better spatial accuracy in the characters (prediction A). Note that the task consisted of reproducing graphic patterns with the dominant hand and in the presence of the model. We suppose that displaying the model allowed the participants to reproduce it accurately from the very first trial. Consequently, the learning consisted more of improving the kinematics than of improving the spatial accuracy, as children do when they learn how to write and free themselves from the models of the characters (Chartrel and Vinter, 2008).

The positive effects of sonification were present when the characters were first learned without then with sonification but not in the reverse order. This order effect, previously observed (Danna et al., 2015b), can be interpreted in the light of the theory of Event Coding (Prinz, 1997; Hommel et al., 2001): When characters have first been learned with sounds, a multimodal (visual, proprioceptive and auditory) representation of the graphic pattern, including the internalized sounds, would have been created. Then, this multimodal representation would be reactivated even if the sounds associated with the movement are no longer supplied.

### In deafferented subjects

Contrary to the control participants who performed better in all post-tests, whatever the sonification condition, the deafferented subjects were unable to learn the characters when training was attempted without sonification. In other words, they were unable to learn new kinematic properties leading to producing fluent graphic patterns whereas the controls were able to do so. This finding strongly suggests that without proprioceptive feedback, motor learning would be either longer or even impossible. This is consistent with the observation that handwriting automaticity in a deafferented patient (GL) was impaired and that proprioception would be a prerequisite to maintain a learned and automated complex motor behavior such as handwriting (Hepp-Reymond et al., 2009). More generally, it has been shown that proprioception plays an important role in the updating of an internal model of limb dynamics used to program motor commands (Sainburg et al., 1995; Krakauer et al., 1999; Pipereit et al., 2006), even if dynamic information may be inferred solely on the basis of vision (Fleury et al., 1995; Sarlegna et al., 2010).

Interestingly, movement sonification seems to be more efficient in deafferented persons than in control participants. In the short-term, the effects of sonification were larger in deafferented, than in control subjects for all kinematical variables (prediction B). More precisely, sonification gave rise to a larger improvement in movement fluency in both deafferented subjects, a larger improvement in velocity for IW than for the controls, and a larger decrease in stops for GL than for the controls. These findings support the hypothesis that translating kinematic information into auditory information substitutes for proprioceptive input. Hearing their sonified movement allowed the deafferented subjects to become informed about the kinematics of their movements that they can no longer feel through proprioception. As GL expressed after the experiment, they “feel their movement by hearing it.” Another, more speculative, hypothesis to explain why deafferented subjects benefited more from the sonification could be that they process auditory information better than controls. It is known that sensory deprivation leads to significant cross-modal brain reorganization which is paralleled by enhanced perceptual abilities. For example, (Bavelier et al., 2006) showed enhancements in visual cognition in deaf subjects due to a reorganization of multisensory areas, highlighting cross-modal interactions as a fundamental feature of brain organization and cognitive processing. The symmetrical effect was observed by (Lessard et al., 1998) who showed that early-blind subjects were able to localize sound sources better than sighted subjects. However, sight and hearing both capture environmental information. Cross-modal enhancements between these two exteroceptive senses when one of them is missing is more likely than enhancements of auditory sensitivity in deafferented subjects although the reverse, enhancement of kinesthetic sensitivity in deaf subjects, has been observed (Levänen and Hamdorf, 2001).

Although the sonification helped the deafferented subjects to learn the new characters in the short term, about 2 h after the training sessions, their performances were similar to those in the pre-test, contrary to the controls who maintained a higher performance. A first hypothesis is that applying auditory information only facilitates the control of ongoing movement in deafferented subjects but does not permit to learn a new motor pattern (prediction C). In other words, in the post-test following the learning sessions with sonification, they wrote better because they kept in short-term memory the movement they had performed just before, but not necessarily because they learned the motor pattern. This hypothesis is supported by their performance during the training sessions: Both deafferented subjects exhibited a fast effect of sonification, from the very first trials, but did not improve over the following repetitions. If this explanation holds, sonification would serve as “sensory prosthesis” helping the deafferented subjects to “feel” (by ear) their movement and to better produce it when the sounds are present, but would not able them to permanently change their motor performance without the prosthesis. Another hypothesis is that producing sonified movement during the training did lead the deafferented subjects to create a multimodal representation which was not maintained over the time in the present experiment because the training period was too short.

### Between deafferented subjects

The initial performance differed between the two deafferented subjects who used opposite strategies. In the pre-test, GL was slower and less fluent than IW, confirming previous observations according to which GL would generally tend to use on-line visual feedback to guide her movement whereas IW would rely on forward motor planning (Cole and Paillard, 1995). These authors reported that both deafferented subjects can write, but their techniques for maintaining accuracy with their eyes shut differed: On the one hand, GL was very slow and, when drawing the letters, she tended to place them in the wrong area of the paper. On the other hand, IW moved fast across the page in an attempt to preserve both shape and correct framing of his writing space, at the cost of accuracy in the shape of the letters. If GL was slower than IW because of a greater reliance on visual control, why was she finally less accurate than him? It is likely that she tended to discretize her movements into many sub-movements (strokes) separated by stops (Ghez et al., 1990) have shown that the spatial accuracy of deafferented subjects was particularly affected at the endpoint of the movement, even under close visual control. We thus suppose that the stops made by GL in order to visually control her movement led her to be *in fine* less accurate.

Consequently, training and sonification had different effects in GL and IW. In GL, the learning curve is comparable to that of the controls (with more variability in her performance), with a greater effect on her. Usually, the poor performance of people beginning to learn to write is the consequence of a close visual control. This visual control gradually decreases with training, paving the way for a more automatic control (Danna and Velay, 2015). It is worth noting that audition is available for the provision of supplementary information during the execution of silent movements, especially in deafferented subjects that use their vision for controlling and adapting their movements. Furthermore, according to the modality appropriateness hypothesis (Welch and Warren, 1980), audition would be more accurate than vision for the treatment of spatiotemporal information about the ongoing movements. Consequently, we hypothesize that training with sonification helped GL to decrease her visual control, leading her to write more fluently thanks to a shift from a *product-oriented* (the written trace) to a *process-oriented* (the movement that generates the trace) control. The initial performance of IW suggest a process-oriented control from the beginning of the learning task. Consequently, sonification during training would not change his initial feedforward strategy but led him to program faster movements to the detriment of spatial accuracy, suggesting a change in speed-accuracy tradeoff. In any case, the opposite results in GL and IW highlight the importance of studying two deafferented subjects to understand the impact of proprioception deprivation on motor control and learning.

### Conclusion and perspectives

This study confirms the potential of movement sonification for motor control and learning. Of course, sonifying the handwriting of people with total proprioceptive loss might appear anecdotal, but it demonstrates that auditory signals may act in substitution of proprioceptive deficit. Clinical applications may be numerous: people with a limb prosthesis, with a stroke, with some peripheral nerve injury, or parkinsonian patients with proprioceptive integration deficits (Schneider et al., 1987; e.g., Klockgether et al., 1995) may potentially be interested. Applied to other human movements, such as walking for example, sonification could be a new “prothestic” device accessible at a much lower cost to millions of people. At a more fundamental level, neuroimaging and EEG studies must be conducted in order to determine the neural basis of auditory-proprioception substitution.

## Ethics statement

This study was carried out in accordance with the recommendations of the Aix-Marseille University and the CNRS (N°; RCB 2010-A00155-34) with written informed consent from all subjects. All subjects gave written informed consent in accordance with the Declaration of Helsinki. The protocol was approved by the Aix-Marseille University and the CNRS&#39.

## Author contributions

Conceived and designed the experiments: JD, JV. Performed the experiments: JD, JV. Analyzed the data: JD. Wrote the paper: JD, JV.

## Funding

This work, carried out within the Labex BLRI (ANR-11-LABX-0036), has benefited from support from the French Government, managed by the French National Agency for Research (ANR), under the project title Investments of the Future A/MIDEX (ANR-11-IDEX-0001-02) and under the CNRS project DEFISENS.

### Conflict of interest statement

The authors declare that the research was conducted in the absence of any commercial or financial relationships that could be construed as a potential conflict of interest.

## Supplementary material

Supplementary Video File 1 **Example of sonified handwriting of a deafferented subject (IW) in eight reproductions of a character during training session with sonification**.

Supplementary Figure 1 **Presentation of the four characters written by the two deafferented subjects (GL and IW) in the pre-test (PRE), in the short-term post-tests following the training phases (POST ST) and in the long-term post-test (POST LT)**. The characters learned with sonification just before the POST ST are surrounded.
