# Supplementary figures and images for: On the Auditory-Proprioception Substitution Hypothesis: Movement Sonification in Two Deafferented Subjects Learning to Write New Characters
Source: Front Neurosci. 2017 Mar 23;11:137. doi: 10.3389/fnins.2017.00137 (PMC5362618; doi:10.3389/fnins.2017.00137)

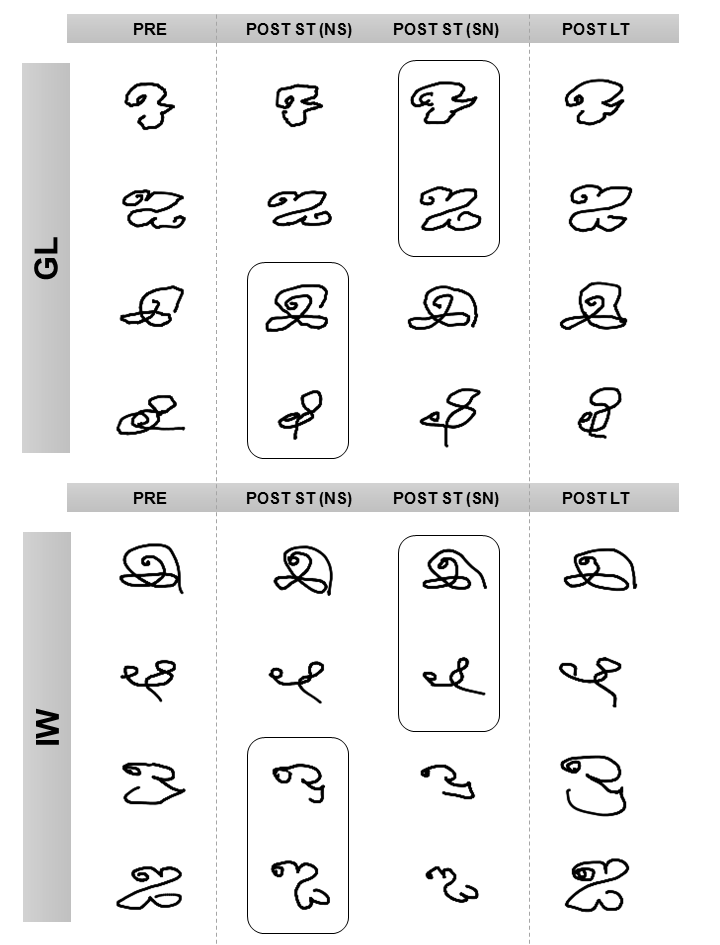

Supplement: Supplementary Figure 1 — Presentation of the four characters written by the two deafferented subjects (GL and IW) in the pre-test (PRE), in the short-term post-tests following the training phases (POST ST) and in the long-term post-test (POST LT). The characters learned with sonification just before the POST ST are surrounded. [file Image1.TIF]
